# Chinese Herbal Medicine on Dyslipidemia: Progress and Perspective

**DOI:** 10.1155/2014/163036

**Published:** 2014-02-13

**Authors:** Ming Guo, Yue Liu, Zhu-Ye Gao, Da-zhuo Shi

**Affiliations:** ^1^Cardiovascular Diseases Center, Xiyuan Hospital, China Academy of Chinese Medical Sciences, Beijing 100091, China; ^2^China Heart Institute of Chinese Medicine, China Academy of Chinese Medical Sciences, Beijing 100091, China

## Abstract

Dyslipidemia is an independent risk factor of cardiovascular diseases. The statins are a milestone in the primary and second prevention of cardiovascular diseases and significantly improved its prognosis. Along with the long-term treatment with statins in combination with other hypolipidemic drugs or alone, its safety has attracted a particular attention in clinic, such as the elevation of transaminase and rhabdomyolysis, which have raised an idea of developing the other types of lipid-lowering agents from botanic materials. Traditional Chinese medicine (TCM) has been used in clinical practice for more than 2000 years in China and showed some beneficial effects for human health and many diseases. Recently, many studies demonstrated a favorable effect of TCM for treating dyslipidemia; however, its mechanism remains unclear or totally unknown. The progress and perspective of studies on dyslipidemia with single Chinese herb and its monomers or effective extracts during the past 10 years are discussed in the present review.

## 1. Introduction

Dyslipidemia is characterized by elevated level of total cholesterol (TC), triglyceride (TG), and low-density lipoprotein cholesterol (LDL-C) and by lowered level of high-density lipoprotein cholesterol (HDL-C) in serum. Dyslipidemia is one of the major independent risk factors for coronary heart disease (CHD) and stroke [1]. The “2013 ACC/AHA Guideline on the Treatment of Blood Cholesterol to Reduce Atherosclerotic Cardiovascular Risk in Adults” emphasized that the progressively regulating dyslipidemia is the pivotal controlling method for risk factors of ischemic cardiovascular events [2]. A large number of evidence indicated that the statins (3-hydroxy-3-methyl-glutaryl-coenzyme reductase inhibitor) significantly reduce the morbidity and mortality of cardiovascular and cerebrovascular events, such as MI and stroke [3]. Along with a long-term use of statins in combination with other hypolipidemic drugs or alone, however, its safety has attached a great concern from scientists and researchers, such as transaminase and creatinine elevation, skeletal muscle pain, and creatine kinase elevation. Therefore, developing novel classes of hypolipidemic agents which possess high efficiency and fewer adverse effects has still been a focus on the treatment of dyslipidemia.

**Figure 1:**
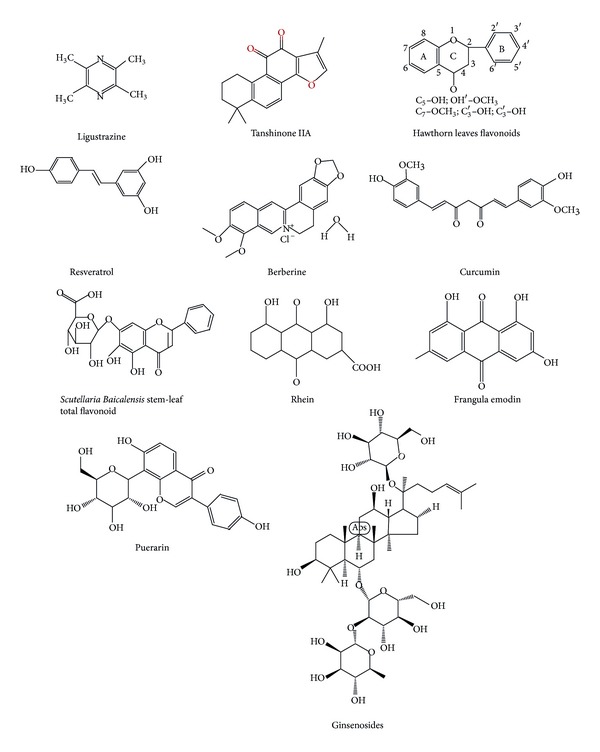
Chemical structures of effective components of Chinese herbs for dyslipidemia.

Although the hyperlipidemia has not been used in traditional Chinese medicine (TCM) term, patients with hyperlipidemia exhibited the similar etiology and pathological changes which charactered as phlegm, dampness, and blood stasis in TCM theory. Moreover, accumulating evidence has indicated that the TCM could improve phlegm, dampness, and blood stasis syndromes manifested in patients with hyperlipidemia even exhibit a beneficial effect for lowering hyperlipidemia [4, 5]. Due to the complicated mechanism of TCM on lipid lowering, most researches currently focus their attention on the effects of Chinese herb monomer or effective extracts in hyperlipidemia (see Figure 1). Studies show that the following Chinese herbs possess a favorable effect on hyperlipidemia to extent degree, which might be classified into four categories: (1) clearing heat and removing toxicity, for example, Radix et Rhizoma Rhei, Rhizoma polygoni cuspidati, Semen Cassia, *Coptis chinensis*, *Scutellaria baicalensis*, *Gynostemma pentaphyllum*, and Radix Puerariae; (2) promoting blood circulation and removing blood stasis, for example, Fructus crataegi, Red yeast rice, Rhizoma, Radix salvia miltiorrhizae, and Turmerone; (3) eliminating dampness and phlegm, for example, Rhizoma alismatis, Plantain seed, and folium nelumbinis; (4) tonifying energy of body (including “*Qi*,” *kidney*), for example, Radix Astragali, Radix Ginseng, and Radix polygoni multiflori (see Table 1). In present review, we summarized the clinical and experimental studies of single Chinese medicine and its monomers or effective extracts on dyslipidemia published during the recent 10 years.

## 2. Single Chinese Herb and Its Monomers or Extracts

### 2.1. Radix Et. Rhizoma Rhei (Da Huang)

Radix et. Rhizoma Rhei is derived from the root and rhizome of Rheum palmatum Linn. (Polygonaceae), Rheum tanguticum Maxim. Ex Balf. (Polygonaceae), or Rheum officinale Baill (Polygonaceae). It is used to purge fire, to remove stagnation by purgation, to cool the blood, to remove toxins, and to remove blood stasis. Anthraquinones, a main active component of Rhubarb, including rhein, aloeemodin, emodin, chrysophanol, and physcion, exhibited lipid-lowering roles by promoting intestinal peristalsis and inhibiting the intestinal absorption of cholesterol [6]. Gao et al. found that rhein, at an oral dosage of 150 mg/kg/day for 12 weeks, was proved to be lowering serum TG, TC, and LDL-c levels in db/db mice with diabetic nephropathy [7]. The powders of rhubarb administrated, at 5 g/day orally for 24 weeks, decreased serum TG and TC levels in patients with diabetic nephropathy [8]. Danthron is another extract of rhubarb, study showing that, at 0.1 *μ*mol/L, 1 *μ*mol/L, and 10 *μ*mol/L of culture medium, dose-dependently promoted the phosphorylation of Adenosine monophosphate activated protein kinase (AMPK) and acetyl-CoA carboxylase (ACC) in both HepG2 and C2C12 cells. Likewise, danthron significantly reduced sterol regulatory element-binding protein 1c (SREBP1c) synthesis and fatty acid synthetase (FAS) gene expressions; both were closed to the lipid metabolism [9]. In addition, Li and Liu found that the powders of rhubarb administrated at 9 g/day for 5 days in 30 health volunteers caused some side-effects, such as vomiting, headache, diarrhea, and abdominal pain [10]. You reported that the decoction boiled from rhubarb, for example, at 8 g, 16 g and 32 g/kg/day for 5 days in mice, caused fatty degeneration of hepatic cell [11].

### 2.2. *Polygonum cuspidatum* (Hu Zhang)

*Polygonum cuspidatum* (PC) is derived from the dried root and rhizome of *Polygonum cuspidatum* Sieb. et Zucc. It dispels wing dampness, removes stagnation, relieves pain, and reduces phlegm. Polydatin and resveratrol, the primary active components of PC, inhibited the cholesterol absorption from intestinal tract [12]. Du et al. found that polydatin administrated at oral dosages of 25 mg, 50 mg, and 150 mg/kg/day for 15–21 days significantly decreased TC, TG, and LDL-c levels and increased TC/HDL-c ratio in hyperlipidemic hamsters and rabbits [12, 13]. Park et al. reported that the *Polygonum cuspidatum* water extract (PCWE) at 5 ug/mL and 40 ug/mL of culture medium reduced the cholesteryl ester formation in human hepatocytes by inhibiting A-cholesterol acyltransferase activity (ACAT) in HepG2 cell *in vitro*, and PCWE at the 40 ug/mL inhibited ACAT activity by 50% [14]. Resveratrol administrated at oral dosages of 30 mg and 70 mg/kg/day for 4 weeks significantly lowered serum lipid, hepatic cholesterol (TC), and TG levels and accelerated the excretion of bile acids in hyperlipidemic rats [15]. In addition, Tong had a case report [16] showing that the oral decoction boiled containing 30 g PC caused gastrointestinal adverse reaction.

### 2.3. Semen Cassia (Jue Ming Zi)

Semen Cassia is the ripe seed of *Senna obtusifolia* Linn. (Fabaceae) or *Cassia tora* Linn. (Leguminosae). It clears away the liver fire to improve eyesight and moistens the intestines to relax the bowels. Proteins and anthraquinone glycosides, the active components of Semen Cassiae, displayed a hypolipidemic effect, mainly due to inhibiting cholesterol absorption, synthesis, and HMG-CoA reductase expression [17]. Lin and Xiong found that the extracts from Semen Cassia administrated at oral dosages of 8 mg, 15 mg, and 25 mg/kg/day for 35 days significantly decreased TC, TG, and LDL-c and increased HDL-c in hyperlipidemic rats [18]. Li et al. showed that the administration with extracts from Semen Cassia for 1-week, at 180 mg/kg/day, significantly decreased the levels of TC, TG, and LDL-c in the mice injected intraperitoneally with 75% fresh yelkfluid [19]. Luo et al. documented that the administration with the total anthraquinone from Semen Cassia, at oral dosages of 0.1 g, 0.2 g, and 0.4 g/kg for 2 months, remarkably reduced the serum concentration of TC, TG in Sprague Dawley (SD) rats administrated with alcohol, at 12.5 mL/kg for the 1st month and 11.25 mL/kg for the 2nd and 3rd month for 2 times a day, for 3 months [20]. Zou and Li suggested that the anthraquinone, Semen Cassiae should be used cautiously because of its potential toxicity [21].

### 2.4. Rhizoma Coptidis (Huang Lian)

Rhizoma Coptidis (RC) is derived from the dried root and rhizome of *Coptis chinensis* Franch., Coptis deltoidea C. Y. Cheng et Hsiao, and *Coptis teeta* Wall. It has the role of clearing away heat, eliminating dampness, purging fire, and removing toxin. Its main components include alkaloid and lignans. Among the alkaloids, the alkaloid berberine is an active component for lipid lowering. Zhou et al. found that berberine administrated, at oral dosages of 75 mg, 150 mg, and 300 mg/kg/day for 16 weeks, had a favorable effect in lowering serum TG, TC, and LDL-c and increasing HDL-c [22]. Chang et al. demonstrated that berberine injected intraperitoneally at 200 mg/kg/day for 16 weeks significantly decreased the serum TC, LDL-c levels and hepatic cholesterol in male SD rats treated with high-fat diet (HFD) for 8 weeks and also upregulated LDLR mRNA expression and suppressed HMGR gene expression [23]. Hu et al. found that Caucasian obese human subjects were given 500 mg berberine orally 3 times a day for 12 weeks showing that the blood lipid was significantly reduced and triglyceride and cholesterol were decreased by 23% and 12.2%, respectively [24]. A meta-analysis concerning 11 randomized controlled trials and 874 participants showed that the berberine produced a significant reduction in TC, TG, and LDL-c [53]. In addition, Zhang et al. reported that berberine at more than 4 g (overdose) resulted in some adverse reactions, such as drug eruption, allergic reactions, dizziness, and shock [54].

### 2.5. *Scutellaria baicalensis* (Huang Qin)

*Scutellaria baicalensis* is derived from the dried root of *Scutellaria baicalensis* Georgi. It clears away heat, eliminates dampness, purges fire, removes toxin, and stops bleeding. Flavonoid compound is an effective lipid-lowering component in *Scutellaria baicalensis* [55]. Liu et al. found that *Scutellaria baicalensis* stem-leaf total flavonoids (SSTF) administrated at oral dosages of 75 mg, 150 mg/kg/day for 25 days in type 2 diabetic rats with hyperlipidemia significantly reduced the serum TG, TC, and LDL-c levels and increased HDL-c [25]. 0.05% *Scutellaria baicalensis* radix extract was added to the diet in hyperlipidemia rats for 4 weeks, showing decrease of TG and TC of the bioflavonoids group [56]. SSTF was administrated at oral dosages of 25 mg, 50 mg, and 100 mg/kg/day in hyperlipidemia rats for 20 days, indicating that SSTF significantly reduced the serum TC, TG, and LDL-c levels and increased HDL-c and the activity of lecithin cholesterol acyltransferase (LCAT) [57].

### 2.6. Gynostemma Pentaphylla (Jiao Gu Lan)

Gynostemma Pentaphylla (GP) is derived from the dried root and rhizome of *Gynostemma pentaphyllum* (Thunb.) Makino. It clears away heat, removes toxin, relieves cough, and eliminates phlegm. Gypenoside is an active component of GP [58]. Studies have found that the lipid-lowering effect of GP was related to inhibiting fat cells producing free fatty acid and synthesizing neutral fat [26, 59, 60]. GP administrated at oral dosages of 250 mg/kg for 4 days significantly reduced TC (by 33%), TG (by 13%), and LDL-c (by 33%) in the obese Zucker fatty diabetic rat model [26]. Zhou et al. established the hyperglycemia rat model with high-fat diet for 6 weeks and then treated them with high dose (200 mg/kg/day) or low dose (50 mg/kg/day) of GP for 4 weeks and founded that GP could decrease the concentration of serum LDL-c, TC, and TG levels remarkably and raise the concentration of HDL-c [27].

### 2.7. Radix Puerariae (Ge Gen)

Radix Puerariae is the dried root of *Pueraria lobata* (Willd.) Ohwi. (Fabaceae). It clears away heat, purges fire, and removes toxin in the theory of Compendium of Materia Medica. Isoflavone is the active compound of Kudzuvine root, such as puerarin, isoflavoues aglycone, daidzin [61]. Yan et al. found that puerarin administrated at oral dosages of 300 mg/kg/day for 4 weeks significantly reduced the serum and hepatic cholesterol levels of hyperlipidemia rats [62]. Experimental hyperlipidemia rats were injected intraperitoneally puerarin (50 mg/kg/day) for 30 days, showing that the plasma TG, TC, and LDL-c significantly reduced and HDL-c increased [28]. Furthermore, oral administration of Kudzuvine root flavones at 100 mg/kg/day for 5 days was reported to enhance hepatic lipid metabolism in ovariectomized rats [29]. Patients with puerarin injections may cause certain adverse effects, such as allergic responses, bloody stool, and backache [63].

### 2.8. Fructus Crataegi (Shan Zha)

Fructus crataegi (FC) is derived from the dried mature fruit of *Crataegus pinnatifida* Bunge. var. major N.E.Br. (Rosaceae) or *Crataegus pinnatifida* Bunge. (Rosaceae). FC is used to dissipate food accumulation, to improve blood circulation, and to disperse blood stasis. Flavonoids and triterpenic acids are the main active hypolipidemic components of FC [64]. FC aqueous extracts given at an oral dosage of 3.6 g/day for 3 months were demonstrated to lower blood TC, TG, and LDL-c in 45 hyperlipidemic volunteers [30]. 80% ethanolic extract administrated at oral dosages of 30, 100 mg/kg/day for 4 weeks in hyperlipidemic rats markedly reversed the increased plasma TC and HDL-c levels [31]. A study on mice that were fed with high-fat diets following the oral administration of FC extracts at a dosage of 250 mg/kg/day for 7 days *in vivo* indicated that FC's lipid-lowering action may be related to increased levels of liver PPAR*α* [65].

### 2.9. Fermentum Rubrum (Red Yeast Rice)

Fermentum Rubrum, popularly known as red yeast rice (RYR) which is the fermented product of Monascus purpureus on rice. It is composed of 13 kinds of natural statins, unsaturated fatty acids, ergosterol, amino acids, flavonoids, alkaloid, trace element, and so forth. 79 patients with baseline LDL-c level of 5.28 mmol/L received a twice daily dose of red yeast rice (600 mg) for 8 weeks in a randomized, double-blind, placebo-controlled study, which found that this therapy could reduce LDL-c by 27.7%, TG by 21.5%, and TC by 15.8% [32]. 72 patients with idiopathic persistent nephritic syndrome with secondary dyslipidemia were randomly given Monascus purpureus Went rice at 600 mg twice one day orally, which significantly reduced serum cholesterol after 6 months and 1 year [33]. XueZhiKang capsule is the extract of red yeast rice. In china, scholars made a systematic review on the clinical randomized controlled trials for treating hyperlipidemia with Xuezhikang, which included 22 randomized trials and a total of 6520 participants, and showed that xuezhikang remarkably lowered TC, TG, and LDL-C compared with theinositol nicotinate [66]. Animal safety evaluations indicate that RYR does not cause any toxic effects in albino rats [67]. However, dyslipidemia patients treated with RYR (1200 mg/day) experienced a few nonserious side effects, such as heartburn, flatulence, dizziness, and gastrointestinal discomfort [63].

### 2.10. Rhizoma Chuanxiong (Chuan Xiong)

Rhizoma chuanxiong (RC) is the dried rhizome of *Ligusticum chuanxiong* Hort. (Umbelliferae). It promotes blood and qi circulation, expels wind, and alleviates pain.

RC contains a variety of esters and alkaloids. Ligustrazine in RC plays an important role in contributing to hypolipidemic effects of RC [68]. Ligustrazine given at an oral dosage of 20 mg, 80 mg/kg/day in atherosclerosis rats decreased TG levels (by 65.2% and 76.7%), TC (by 53.2% and 77.9%), and LDL-c (by 71.2% and 79%) levels [34]. Tetramethylpyrazine administered at 75 mg, 150 mg/kg/day for 12 weeks in atherosclerosis rabbits, significantly reduced the serum TC, TG, and LDL-c levels [35]. The oral administration of RC causes headaches and injection of ligustrazine can also cause bleeding and allergic responses in certain cases [69].

### 2.11. Radix Salviae Miltiorrhizae (Dan Shen)

Radix Salviae Miltiorrhizae (RSM) is derived from the root and rhizome of *Salvia miltiorrhiza* Bge. (Lamiaceae). It removes blood stasis and promotes blood circulation, relieves pain, regulates menstruation, removes heat from the heart, and relieve restlessness. Dan Shen is widely used to treat patients with coronary artery disease in China. Tanshinone is the main effective component in RSM [70]. Aqueous extracts of RSM given at oral dosages of 50 mg, 100 mg, and 150 mg/kg/day for 4 weeks significantly decreased TC and TG levels and increased HDL-C serum levels in hyperlipidemic rats [36]. Tanshinone IIA (T-IIA) sulfonate intravenous injected (80 mg dissolved in 250 mL 0.9% salt water) at 80 mg/day for 14 days in patients with diabetes mellitus decreased the serum TG, TC, and LDL-c obviously [37]. In addition, human HepG2 cells treated with T-IIA for 24 h exerted a dose-dependent inhibitory effect on ApoB secretion together with triglyceride [71]. RSM may cause abdominal discomfort following long-term administration and also results in internal tissue bleeding when used in combination with aspirin or warfarin [72].

### 2.12. Rhizoma Curcumae Longae (Jiang Huang)

Rhizoma curcumae longae (RCL) is derived from the root and rhizome of *Curcuma longa* L. It removes blood stasis, promotes the circulation of Qi, regulates menstruation, and relieves pain. Curcumin is the main component in RCL [73]. Curcumin (0.05 g/100 g diet) supplementation on a high-fat diet (10% coconut oil, 0.2% cholesterol, wt/wt) fed to hamsters for 10 weeks significantly lowered the levels of free fatty acid (FFA), TG, TC, and LDL-c and elevated the levels of HDL-c and apolipoprotein (apo) A-I and paraoxonase activity in plasma [38]. Curcumin administrated at dosages of 40 mg, 80 mg, and 160 mg/kg/day for 4 weeks in hyperlipidemia rats significantly reduced the serum and hepatic TC, TG, and FFA and increased the HDL-c [39]. *In vitro*, curcumin at 5 uM concentration completely prevented LDL oxidation by CuS0_4_ [74]. The curcumin acting on the low density lipoprotein receptor (LDLR) expression which is measured by Fluo-Microscopy and Fluorescence Flow Cytometric Methods in HepG2 cell obviously upregulated the expression of LDLR [75].

### 2.13. Rhizoma Alismatis (Ze Xie)

Rhizoma alismatis (RA) is derived from the dried stem tuber of *Alisma orientale* (Sam.) Juzep. (Alismataceae). RA promotes diuresis to resolve dampness and expel heat. Triterpenes are the main active components from RA, which exerts its hypolipidemic effects by inhibiting the absorption and synthesis of cholesterol and improving lipid metabolism [76]. The powders of RA administered at oral dosages of 10 g/day for 2 weeks in healthy volunteers reduced the TC, LDL-C, and TG [40]. The oral administration of aqueous and alcoholic RA extracts at 0.3 mL/day for 21 days resulted in significant decreasing in serum TG, and TC, while increased the HDL-c and improved the artheriosclerosis index (AI) in hyperlipidemia SD rats [41]. The adverse effects of RA are correlated with hepatotoxicity following over dosage [42].

### 2.14. Semen Plantaginis (Che Qian Zi)

Semen plantaginis is the ripe seed of *Plantago asiatica* L. or *Plantago depressa* Willd. Semen plantaginis clears heat, causes dieresis, excretes dampness, improves eyesight, and eliminates phlegm. The polysaccharides of Semen plantaginis (PSP) not only have the aperients effect but also the lipid-lowering role [77]. In a multicenter, double-blind, placebo-controlled, parallel, and randomized trial conducted in primary care-clinics in Spain, France, and Holland, mild-moderate hypercholesterolaemic patients (age range: 43–68 years) received 14 g/d of the soluble fibre Plantago ovate (PO)-husk (*n* = 126) for 8 weeks. Po-husk reduced plasma LDL-C by −6%, total cholesterol (TC) by −6%, triglycerides (TG) by −21.6%, and apolipoprotein (Apo) B-100 by −6.7% [78]. Wang et al. found that Plantain seed at dosage of 15 g/kg for 12 weeks can decrease content of lipid and strengthen superoxide dismutase (SOD) activity [43]. Plantain seed administered at oral dosages of 7.5 g, 10 g/100 g for 4-weeks in male Hartley guinea pig significantly reduced the level of TC and LDL-c [44].

### 2.15. Folium Nelumbinis (He Ye)

Folium Nelumbinis is the dried leaf of *Nelumbo nucifera* Gaertn. It is used to clear away summerheat, to lift the lucid yang, to cool the blood, and to stop bleeding. The total alkaloids and flavonoids in Lotus leaves are the main active components of He Ye [79]. Aqueous extracts of Lotus leaves administered at an oral dosage of 400 mg/kg/day for 6 weeks were demonstrated to lower serum TC, TG, and LDL-C levels in rats fed a high-fat diet [45]. The flavonoids extracts of Lotus (50 mg and 200 mg/kg) were orally administered once a day for 28 days in rats, showing that the serum TC, TG, and LDL-c levels were significantly decreased, whereas serum HDL-c level was increased [46]. As demonstrated in the livers of mice that were fed high-fat diets, the mechanisms of action of Lotus leaves may be associated with suppressed expression of FAS, acetyl-CoA carboxylase, and HMG-CoA reductase and the increased phosphorylation of AMP-activated protein kinase [47].

### 2.16. Radix Astragali (Huang Qi)

Radix Astragali is the dried root of *Astragalus propinquus* (Fisch.) Bge. var. mongholicus (Bge.) Hsiao (Fabaceae) or Astragalus membranaceus (Fish.) Bge. (Fabaceae). It replenishes the qi to consolidate superficies and promotes diuresis to relieve edema. MMR polysaccharides, flavonoids, and sponins are the main active components of membranous milkvetch root (MMR) [48]. Astragalus Mongholicus extracts at 0.4% and 0.8% for 5 weeks in rats maintained on a high-cholesterol diet significantly reduced the serum of TG, TC, and LDL-c levels and increased the HDL-c levels and reduced levels of lipid peroxidation [80]. Polysaccharides from Astragalus administered at an oral dosage of 40 mg, 100 mg/kg/day in hyperlipidemia rats for 40 days obviously reduced the serum TC, TG, LDL-c, and MDA levels and increased HDL-c levels [81]. The hypolipidemic mechanisms of MMR polysaccharides *in vivo* may be associated with the increased expression of LDLR and 7-hydroxylase mRNAs and the decreased expression of HMG-CoA reductase mRNA in the liver [82]. The injection of Radix Astragali may cause nausea and allergic response [83].

### 2.17. Radix Ginseng (Ren Shen)

Radix Ginseng is derived from the dried root and rhizome of *Panax ginseng* C.A. Mey. (Araliaceae). It reinforces vital energy, restores the pulse, treats exhaustion, reinforces the spleen to benefit the lungs, promotes the production of body fluids, and calms the mind. Ginseng saponins and polysaccharides are the main active components of Radix Ginseng [84]. Ginseng saponins intragastric administered at an oral dosage of 2 mg/kg/day for 90 days in C57/BL-ApoE gene knockout hyperlipidemia rats can reduce the levels of plasma TC, TG, and LDL-c [49]. Ginseng saponin is divided into Rb1, Rb2, RC, Rd, Re, and Rl [85]. Ginseng saponin Rb administered at an oral dosage of 50 mg, 100 mg, and 200 mg/kg/day in hyperlipidemia rat for 12 days significantly reduced the TG, TC, and LDL-c levels in serum and liver [50]. In addition, Compound k (CK) is a major intestinal metabolite of ginsenosides derived from ginseng radix. *In vitro*, CK significantly activated the AMP-activated protein kinase (AMPK) to affect the lipid metabolism in insulin-esistant HepG2 human hepatoma cells [86]. Ginseng saponins have poor bioavailability following oral administration. Although Ginseng is very safe for oral administration, an overdose or long-term administration of Ginseng may cause the neurotoxicity, cardiotoxicity or allergic reaction [69].

### 2.18. Radix Polygoni Multiflori (He Shou Wu)

Radix Polygoni Multiflori (RPM) is derived from the dried root tuber of *Fallopia multiflora* Thunb. (Polygonaceae). RPM has been used in both raw and prepared pharmaceutical forms. Raw RPM prevents the recurrence of malaria, eliminates toxic materials, moistens the intestine, and relaxes the bowels. Prepared RPM blackens the hair and beard, strengthens the muscles and bones, improves the essence of the blood, and nourishes the liver and kidneys. RPM exerts its hypolipidemic effects primarily by targeting the gastrointestinal tract and inhibiting the absorption of cholesterol [87]. RPM extract administered at an oral dosage of 12 mg and 24 mg/kg/day for 4 weeks in hyperlipidemic rats reduced the serum levels of TC, TG, and LDL-c [51]. Wang et al. found the ethl acetate extracting fraction (EAEF) and stilbene glycoside from the tube of Polygonum multiflorum administered orally at dose of 30 and 60 mg/kg/day for 28 days could reduce the serum TC, TG, and LDL-c levels in hyperlipidemia rats [52]. As demonstrated in experiments with Bel-7402 cells, stilbene glucoside may be a key active component of RPM and involved in both inhibiting cholesterol synthesis and increasing the expression of low-density lipoprotein receptor (LDLR) mRNA [88]. Li et al. found that RPM extracts could regulate the lipid content within liver cell better than RPMP (Radix Polygoni Multiflori Praeparata), but RPMP displayed better effects than RPM in lipid regulation in the circulatory system [89]. Clinical reports have revealed that RPM exhibits hepatotoxicity, allergic responses, and gastrointestinal hemorrhage following chronic treatment [90, 91].

## 3. Perspective

During the past 10 years, the studies on lipid-lowering therapy with Chinese herbs have achieved many progresses to some extent, but some limits are also existed: (1) although the effects of Xuezhikang (extract of red yeast rice) on lowering cholesterol and LDL-c were evidenced in multicenter, large sample, and randomized clinical trials [66, 92], most clinical trials on dyslipidemia with TCM did not show enough power to identify the definite effects due to small samples or unemployment of multicenter, large samples, and randomized design; (2) because of very complicated compounds contained in one herb, even in an extract of one herb, it is a very tough work to clarify the mechanism of TCM for treating dyslipidemia and interaction with western medicines, which lead to some obstacles in clinical application in combination with statins or other chemical agents; and (3) due to different herb has different active compound and different property, which has been taken as *Han* (Cold), *Re* (Heat), *Wen* (Warm) and *Liang* (Cool) according to TCM theory, it is hard in clinical practice to optimize its benefit effects or reduce adverse effects for patients with hyperlipidemia.

Along with a long-term use of statins in combination with other hypolipidemic drugs or alone, the adverse reactions frequently occurred about statins at domestic or abroad. TCM has been widely used in China for more than 2000 years. Screening highly efficient hypolipidemic agents from TCM with fewer adverse effects has attracted more attention, and the mechanisms of TCM for hyperlipidemia become a hot topic in cardiovascular diseases research field recently.

As mentioned above, the TCM has some beneficial effects on the treatment of patients with dyslipidemia and has less adverse effects compared with chemical agents. The advantages and disadvantages of TCM, however, needed to be confirmed in the future clinical trials according to the concept of evidence based medicine. Along with the development of modern scientific techniques, which can be applied in the TCM studies, it is becoming easier to identify how many component one herb contained and which component is a main component for treating dyslipidemia. As we all know, the TCM was used in clinical practice in the formula manner and demonstrated that many formulas and herbs have some favorable effects for dyslipidemia. Therefore, to develop new agents with effectiveness and safety from TCM is a promising way for prevention and treatment of patients with dyslipidemia and even then with cardiovascular diseases.

## Figures and Tables

**Table 1:** The most frequently used single Chinese herbs for dyslipidemia.

Number	Herbs	Dosage/administration/time	Effects	Components	References
1	Radix et. Rhizoma Rhei (Dahuang)	Human: powder, 5 g/day, Po, 24 weeks; Db/db mice: rhein, 150 m/kg/day, Po, 12 weeks	TG↓ TC↓ LDL-C↓	Anthraquinones	[7, 8]
2	Rhizoma ploygoni cuspidate (Hu Zhang)	Rabbits: polydatin, 25 mg, 50 mg, 100 mg/kg/day Po, 3 weeks; SD rats: resveratrol 30, 70 mg/kg Po, 4 weeks	TG↓ TC↓ LDL-C↓	Polydatin, resveratrol, and emodin	[12, 13, 15]
3	Semen Cassia (Jumingzi)	SD rats: extracts, 8, 15, 25 mg/kg Po, 35 days; SD rats: anthraquinones, 0.1, 0.2, 0.4 g/kg Po, 2 months	TG↓ TC↓ LDL-C↓	Anthraquinones, protein	[18, 20]
4	*Coptis chinensis* (Huanglian)	Human: berberine, 500 mg tid Po, 12 weeks; SD rats: berberine, 200 mg/kg/day ip, 16 weeks; SD rats: berberine 75 mg, 150 mg, 300 mg/kg/day po 16 weeks	TC↓ LDL-C↓ TG↓ HDL-c↑	Alkaloid berberine	[22–24]
5	*Scutellaria baicalensis* (Huangqin)	SD rats: SSTF, 75 mg, 150 mg/kg Po, 25 days;	TC↓ LDL-C↓ TG↓ HDL-c↑	Flavonoid	[25]
6	*Gynostemma pentaphylla* (Jiaogulan)	Mice: powder, 250 mg/kg, Po, 4-day; SD rats: extract, 50 mg, 200 mg/kg/day, Po, 4 weeks	TG↓ TC↓ LDL-C↓	Gypenoside	[26, 27]
7	Radix Puerariae (Gegen)	Wistar rats: puerarin, 50 mg/kg/day ip, 30 days; ovariectomized rats: flavones, 100 mg/kg/day, po, 5 weeks	TC↓ LDL-C↓ TG↓ HDL-c↑	Puerarin	[28, 29]
8	Fructus crataegi (Shan zha)	Human: aqueous extracts, 3.6 g/kg, Po, 3 months; rats: ethanol extracts, 30 mg, 100 mg/kg/day. Po., 4 weeks	TG↓ TC↓ LDL-C↓	Flavonoids, triterpenic acids	[30, 31]
9	Red yeast rice (Hongqu)	Human: rice, 600 mg/day, Po, 8 weeks; human: rice, 1.2 mg, po, 6 months–1 year	TC↓ LDL-C↓ TG↓	Lovastatin, sterols, Isoflavones and isoflavone glycosides, and MUFA	[32, 33]
10	Rhizoma chuanxiong	Rats: ligustrazine, 20 mg, 80 mg/kg, Po, 6 weeks; rabbits: ligustrazine, 75 mg, 150 mg/kg/day, Po, 12 weeks	TG↓ TC↓ LDL-C↓	Lactones, total alkaloids	[34, 35]
11	Radix salvia miltiorrhizae (Danshen)	Rats: extracts, 50, 100, 150 mg/kg/day, Po, 4 weeks; human: tanshinone IIA, 80 mg/day, ivgtt. 14 days	TC↓ LDL-C↓ TG↓ HDL-c↑	Tanshinone IIA	[36, 37]
12	Turmerone (Jianghuang)	Hamsters: curcumin, 0.05 g/100 g, Po, 10 weeks; SD rats: curcumin, 40, 80, 160 mg/kg, Po, 4 weeks.	TC↓ LDL-C↓ TG↓ FFA↓ HDL-c↑	Curcumin	[38, 39]
13	Rhizoma alismatis (Zexie)	Human: powders, 10 g/day, Po, 2 weeks; SD rats: extracts, 0.3 mL/day, Po, 21 days.	TC↓ LDL-C↓ TG↓	Triterpenes	[40, 41]
14	Plantain seed (Cheqianzi)	Human: polysaccharides, 14 g/day, Po, 8 weeks; rats: powder, 15 g/kg, Po, 12 weeks; pig: plantain seed, 7.5, 10 g/100 mg, po, 4 weeks.	TC↓ LDL-C↓ TG↓	Polysaccharides	[42–44]
15	Folium nelumbinis (Heye)	SD rats: aqueous extracts, 400 mg/kg/day, Po, 6 weeks; mice: flavonoids, 50, 200 mg/kg/day, Po, 28 days.	TC↓ LDL-C↓ TG↓	Total flavonoids, alkaloid	[45, 46]
16	Radix Astragali (Huangqi)	Rat: extracts, 0.4%, 0.8%, Po, 5 weeks; rat: polysaccharides, 40, 100 mg/kg/day, Po, 40 days.	TC↓ LDL-C↓ TG↓ HDL-c↑	Polysaccharides, flavonoid, and saponin	[47, 48]
17	Radix Ginseng (Renshen)	Mice: ginsenoside, 2 mg/kg/days, Po, 90 days; rats: ginsenoside Rb, 50 mg, 100 mg, 200 mg/kg/day, Po, 12 days	TC↓ LDL-C↓ TG↓ HDL-c↑	Ginsenoside, ginseng, and polysaccharides	[49, 50]
18	Radix Polygoni Multiflori (Heshouwu)	Rats: extracts, 12, 24 mg/kg/day, Po, 4 weeks; rats: EAEF, 30, 60 mg/kg/day, Po, 28 days.	TC↓ LDL-C↓ TG↓ HDL-c↑	Anthraquinones, polysaccharides	[51, 52]
